# Dataset on the conceptual evaluation of carbon capture rates using Ca(OH)_2_ in the calcium looping process

**DOI:** 10.1016/j.dib.2025.111415

**Published:** 2025-02-25

**Authors:** Markus Secomandi, Kari Myöhänen, Jouni Ritvanen

**Affiliations:** Lappeenranta-Lahti University of Technology, LUT School of Energy Systems, P.O. Box 20, FI-53851, Lappeenranta, Finland

**Keywords:** Decarbonisation, Modelling, Calcium hydroxide, 1.5D model

## Abstract

Carbon capture in industrial processes is essential for mitigating climate change, especially in energy-intensive sectors like cement and power generation. Among the available carbon capture technologies, calcium looping (CaL) stands out as a feasible post-process solution, which typically captures around 90% of emitted CO₂. However, injecting calcium hydroxide (Ca(OH)₂) can increase capture rates to about 99%. This dataset illustrates how introducing Ca(OH)₂ can help trap more carbon dioxide in industrial systems, highlighting the importance of key factors such as the injection rate and the reactor entry point. By offering detailed simulations under various conditions, the dataset serves as a resource for engineers and researchers looking to reduce emissions in challenging industrial environments and develop new models to support process design.

The dataset consists of results from a series of 79 simulated cases evaluating the effect of Ca(OH)₂ injection on carbon capture efficiency in the calcium looping process. The simulations were performed using a 1.5D reactor model developed in the Matlab/Simulink environment based on the configuration of the La Pereda pilot plant.

The dataset includes Excel files containing detailed case-specific data, including all the solved parameters, such as carbon capture efficiencies, heat balances, reactor temperature profiles, solid circulation rates, gas flow compositions, and the distribution of char, sorbent, and fine Ca(OH)₂ particles. Variables related to the Ca(OH)₂ injection, such as injection flow rate and elevation, are also included to allow examination of their effects on the system. The key parameters are visualized within the files.

This dataset provides a valuable resource for researchers working on carbon capture technologies, reactor modelling, and process optimisation. It can be used to validate new models, explore alternative configurations of calcium looping systems, or investigate the interaction of injected Ca(OH)₂ with other system variables. The dataset is particularly relevant for applications involving calcium looping by circulating fluidised bed reactors and high CO₂ capture requirements.

Specifications Table

**Table:** 

Subject	Engineering & Materials science
Specific subject area	Carbon capture and storage, calcium looping, reactor modelling, and process optimisation in circulating fluidised bed systems.
Type of data	Table (.xlsx format)
Data collection	The dataset was generated using a 1.5D reactor model, which is presented in detail in the related research article [1]. The model simulated the calcium looping process based on the La Pereda pilot plant configuration [2,3]. Input parameters included reactor dimensions, gas and solid flow rates, and injection conditions for Ca(OH)₂. Conservation equations for mass, energy, and species were solved using Simulink ODE solvers. Data include reactor temperature, pressure, gas composition, solid flow rates, and carbonation profiles along the reactor height, calculated under steady-state conditions.
Data source location	Institution: LUT University City/Town/Region: Lappeenranta Country: Finland Latitude and longitude: 61°03′55.0"N 28°05′27.0"E
Data accessibility	Repository name: Zenodo Data identification number: DOI: 10.5281/zenodo.14671741 Direct URL to data: https://zenodo.org/records/14671742
Related research article	M. Secomandi, M. Nikku, B. Arias, J. Ritvanen. A conceptual evaluation of the use of Ca(OH)_2_ for attaining carbon capture rates of 99% in the calcium looping process. Int. J. Greenh. Gas Control, 139 (2024), article 104279. https://doi.org/10.1016/j.ijggc.2024.104279.

## 1 Value of the Data

• The dataset provides detailed simulation results for 79 cases with different settings, capturing key variables such as reactor temperature profiles, gas and solid compositions, and carbon capture efficiencies in the calcium looping process. These detailed data can assist researchers in understanding and modelling complex calcium looping reactor systems.
• The data include the effects of Ca(OH)₂ injection on reactor performance, providing insights into the enhancement of carbon capture efficiency. This information is valuable for developing and validating advanced calcium looping models.
• Researchers can reuse the dataset to explore alternative configurations, refine reactor designs, or compare experimental results with validated model outputs, aiding in process optimisation for novel carbon capture systems.
• The dataset supports the development of new modelling frameworks or methodologies for simulating circulating fluidised bed reactors, especially for applications requiring high CO₂ capture rates.
• The data enable sensitivity analyses of critical parameters, such as injection rates and elevations, enhancing the understanding of their influence on reactor performance in carbon capture applications.

## 2 Background

The dataset was generated to evaluate the potential of calcium hydroxide (Ca(OH)₂) injection to enhance the carbon capture efficiency of the calcium looping process. The simulations were conducted using a 1.5D reactor model developed to study the relationships between key reactor parameters, such as carbon capture, temperature profiles, solid circulation rates, and gas and solid compositions, in a calcium looping system. The dataset is based on the configuration and operating conditions of the La Pereda pilot plant, providing realistic case studies for reactor modelling.

The dataset complements the original research article by offering detailed simulation outputs, which explore the effects of varying injection flow rates, injection elevations, and char combustion profiles on carbon capture efficiency. These files include all modelled parameters, offering a valuable resource for researchers seeking to replicate, validate, or extend the original findings. They also enable further investigation into reactor design and optimisation strategies for high CO₂ capture rates*.*

## 3 Data Description

The dataset consists of 79 Excel files, each representing a single simulated case. The data used in the original publication are listed in Table 1, where the ending of each filename is given. To obtain the full filename, simply append the corresponding ending found below to ‘CaL1D_results’. For example, data on the cooled case with no char combustion is found in the file ‘CaL1D_results_cool.xlsx’. The dataset also contains 10 additional files containing draft calculations that were not utilised in the actual study.

**Table 1:** The various cases simulated for the original paper [1] and their corresponding Excel files.

	Char combustion profile		
	None	400µm	150µm
Base case	1	1	1
	_this	_this_CO_this	_this_COfinest_this
Cooled case, additional cooling surfaces	1	1	1
	_cool	_this_CO_cool	_this_COfinest_cool
Cooled case, increased internal circulation	1	-	-
	_this_circcool2	-	-
Hydroxide	24	24	24
- molar ratio = 1; h_inj = 3 m	_hydrox_1_3	_this_CO_hydr1_3	_this_COfinest_1_3
- molar ratio = 1; h_inj = 4.5 m	_hydrox_1_45	_this_CO_hydr1_45	_this_COfinest_1_45
- molar ratio = 1; h_inj = 6 m	_hydrox_1_6	_this_CO_hydr1_6	_this_COfinest_1_6
- molar ratio = 1; h_inj = 7.5 m	_hydrox_1_75	_this_CO_hydr1_75	_this_COfinest_1_75
- molar ratio = 1; h_inj = 9 m	_hydrox_1_9	_this_CO_hydr1_9	_this_COfinest_1_9
- molar ratio = 1; h_inj = 10.5 m	_hydrox_1_105	_this_CO_hydr1_105	_this_COfinest_1_105
- molar ratio = 1; h_inj = 12 m	_hydrox_1_12	_this_CO_hydr1_12	_this_COfinest_1_12
- molar ratio = 1; h_inj = 13.5 m	_hydrox_1_135	_this_CO_hydr1_135	_this_COfinest_1_135
- molar ratio = 2; h_inj = 3 m	_hydrox_2_3	_this_CO_hydr2_3	_this_COfinest_2_3
- molar ratio = 2; h_inj = 4.5 m	_hydrox_2_45	_this_CO_hydr2_45	_this_COfinest_2_45
- molar ratio = 2; h_inj = 6 m	_hydrox_2_6	_this_CO_hydr2_6	_this_COfinest_2_6
- molar ratio = 2; h_inj = 7.5 m	_hydrox_2_75	_this_CO_hydr2_75	_this_COfinest_2_75
- molar ratio = 2; h_inj = 9 m	_hydrox_2_9	_this_CO_hydr2_9	_this_COfinest_2_9
- molar ratio = 2; h_inj = 10.5 m	_hydrox_2_105	_this_CO_hydr2_105	_this_COfinest_2_105
- molar ratio = 2; h_inj = 12 m	_hydrox_2_12	_this_CO_hydr2_12	_this_COfinest_2_12
- molar ratio = 2; h_inj = 13.5 m	_hydrox_2_135	_this_CO_hydr2_135	_this_COfinest_2_135
- molar ratio = 3; h_inj = 3 m	_hydrox_3_3	_this_CO_hydr3_3	_this_COfinest_3_3
- molar ratio = 3; h_inj = 4.5 m	_hydrox_3_45	_this_CO_hydr3_45	_this_COfinest_3_45
- molar ratio = 3; h_inj = 6 m	_hydrox_3_6	_this_CO_hydr3_6	_this_COfinest_3_6
- molar ratio = 3; h_inj = 7.5 m	_hydrox_3_75	_this_CO_hydr3_75	_this_COfinest_3_75
- molar ratio = 3; h_inj = 9 m	_hydrox_3_9	_this_CO_hydr3_9	_this_COfinest_3_9
- molar ratio = 3; h_inj = 10.5 m	_hydrox_3_105	_this_CO_hydr3_105	_this_COfinest_3_105
- molar ratio = 3; h_inj = 12 m	_hydrox_3_12	_this_CO_hydr3_12	_this_COfinest_3_12
- molar ratio = 3; h_inj = 13.5 m	_hydrox_3_135	_this_CO_hydr3_135	_this_COfinest_3_135

**Fig. 1:**
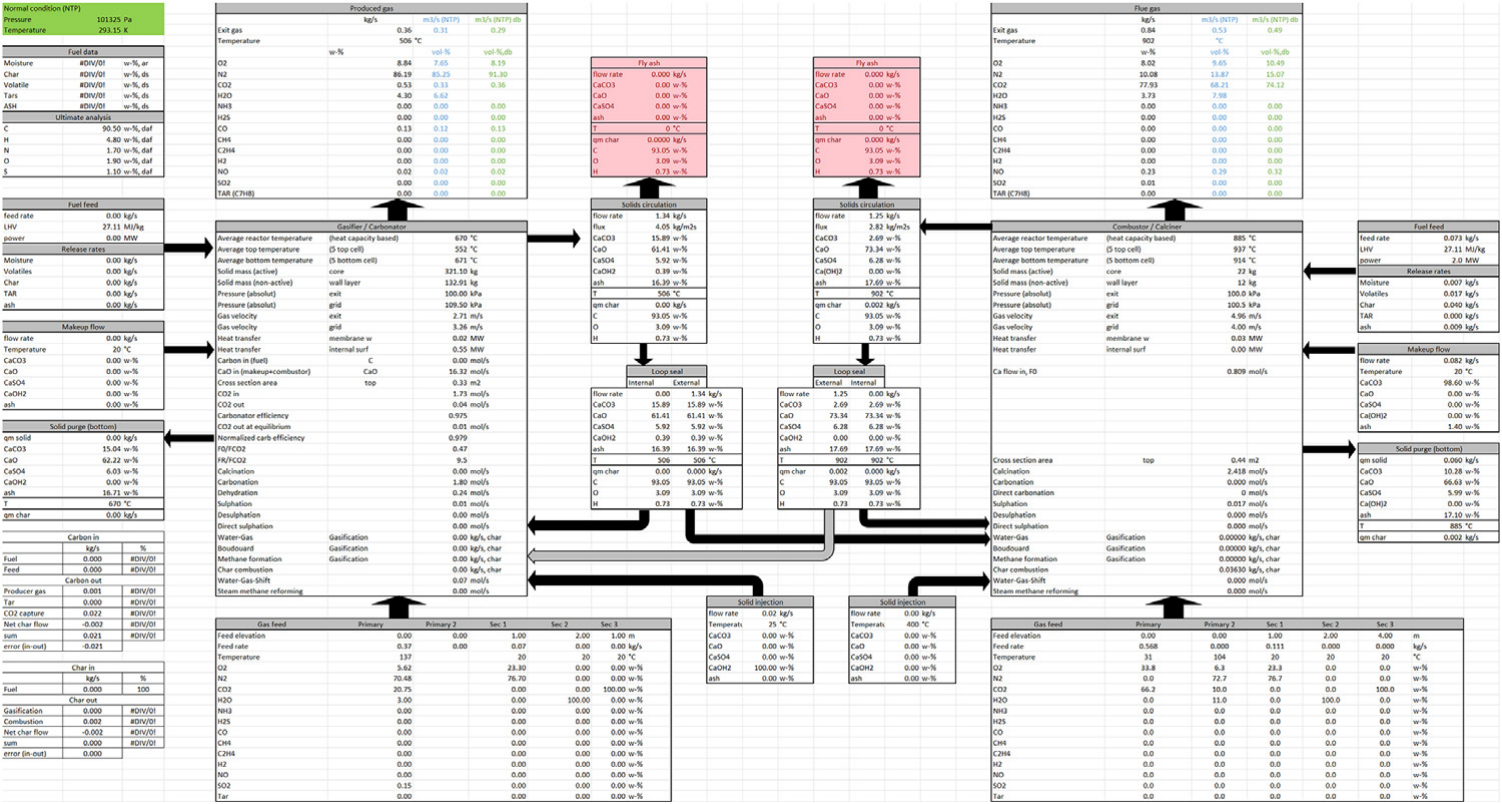
Example of 0D-results.

All Excel files are structured in the same way. The ‘Model data’ sheet contains tabled 1D profiles of the most important variables, such as gas and solid compositions, flow rates, temperatures, densities, and reaction rates. The ‘Inputs’ sheet contains tables with information on the different mass flows entering the reactors, whereas the ‘Mass-balances’ and ‘Energy-balances’ sheets contain mass and energy balances. The ‘Fuel-balance’ sheet describes the decomposition of fuel into volatiles and char along with their respective compositions. The ‘0D-results’ sheet presents the main process variables, such as flows entering and exiting each reactor, in a clear diagram (Fig. 1). The remaining sheets consist of graphs of various profiles such as pressures, temperatures, compositions and reaction rates.

## 4 Experimental Design, Materials and Methods

### 4.1 Experimental design

The dataset was generated through simulations using a 1.5D reactor model implemented in the Matlab/Simulink environment. The model was applied to evaluate the effect of calcium hydroxide (Ca(OH)₂) injection on carbon capture efficiency in the calcium looping process, focusing on reactor conditions similar to the La Pereda pilot plant configuration. The simulations covered 79 cases, varying key parameters such as injection flow rate, injection elevation, and char combustion profiles. The latest pilot tests involving Ca(OH)₂ injection suggest that high CO₂ capture efficiencies are achievable in practice. The measurement data are currently being processed and will be used to validate the model once fully compiled.

### 4.2 Reactor model description

The 1.5D reactor model simulates circulating fluidised bed reactors and incorporates the following:

• Vertical Segmentation: Reactors are divided into finite volumes along their height to solve mass, energy, and species conservation equations.
• Radial Segmentation: Each vertical segment is split into a core region and a wall layer to represent internal solids circulation.
• Chemical Reactions: Includes reactions for carbonation, calcination, char combustion, and the dehydration-carbonation of Ca(OH)₂.

### 4.3 Software and tools

• Simulink: Used for implementing and solving the model's ordinary differential equations (ODEs) for transient and steady-state conditions.
• MATLAB: For parameter input, result processing, and exporting data to Excel files.

### 4.4 Key assumptions and parameters

1. Sorbent and Injection:
  • Make-up flow was calcitic limestone (98.6% CaCO_3_) fed into the calciner.
  • Ca(OH)₂ was injected into the carbonator as fine (10 µm) particles.
  • Injection elevation varied between 3 and 9 meters above the reactor grid.
2. Operating Conditions:
  • Carbonator and calciner heights: 15 m.
  • Carbonator gas velocity: 3.4 m/s.
  • Temperatures: Carbonator (550–650°C), Calciner (880–915°C).
3. Char Combustion:
  • Two particle size profiles were simulated (150 µm and 400 µm).
  • CO₂ formation rates from char combustion were varied to simulate different combustion rates.

### 4.5 Data acquisition process

1. Base case validation: The model was validated against experimental data from the La Pereda pilot plant. This ensured an accurate representation of reactor behaviour, including temperature and pressure profiles and gas and solid compositions.
2. Parameter variation: Key parameters, such as Ca(OH)₂ injection rates (molar ratios of 1–3) and elevations, were systematically varied to generate a comprehensive dataset.
3. Simulations: Each case was run under steady-state conditions to obtain reactor profiles for temperature, pressure, gas composition, solid flow, and carbon capture efficiency.

### 4.6 Output data

For each case, the following data were exported to Excel files solved along the reactor height and for the reactor outlet:

• Pressure.
• Temperature.
• Gas phase composition.
• Solid phase composition, including CaO, CaCO₃, and char fractions.
• Carbonation rates and equilibrium conditions.

Additionally, the overall results such as total mass and heat balances of both reactors were reported.

## Limitations

None

## Ethics Statement

The authors assure that the work and manuscript follow and meet the ethical requirements of the journal. The work does not involve human subjects, animal experiments, or any data collected from social media platforms.

## CRediT Author Statement

**Markus Secomandi:** Writing – original draft, Writing – review & editing, Visualization, Validation, Software, Methodology, Formal analysis, Conceptualization. **Kari Myöhänen:** Writing – original draft, Writing – review & editing, Data curation. **Jouni Ritvanen:** Supervision, Software, Methodology, Funding acquisition.

## Data Availability

ZenodoDataset on the conceptual evaluation of carbon capture rates using Ca(OH)2 in the calcium looping process (Original data).
